# Sexually dimorphic developmental changes in rat spinal cord pain pathways following neonatal inflammation

**DOI:** 10.14814/phy2.70670

**Published:** 2025-12-19

**Authors:** Kateleen E. Hedley, Annalisa Cuskelly, Rikki K. Quinn, Robert J. Callister, Deborah M. Hodgson, Melissa A. Tadros

**Affiliations:** ^1^ School of Biomedical Sciences & Pharmacy University of Newcastle and Hunter Medical Research Institute Newcastle New South Wales Australia; ^2^ Laboratory of Neuroimmunology, School of Psychology University of Newcastle and Hunter Medical Research Institute Newcastle New South Wales Australia

**Keywords:** astrocytes, cytokines, dorsal root ganglai, lipolysaccharide, Microglia

## Abstract

Early‐life inflammation has a long‐lasting impact on pain behaviors, with neonatal inflammation resulting in altered pain behaviors throughout life. Possible mechanisms underlying these changes lie within the first and second order neurons in the pain neuroaxis. We investigated neuroinflammatory markers in dorsal root ganglia (DRGs) and spinal cords (SC) of Wistar rats (both sexes) following neonatal injection with either LPS or saline (postnatal days (P) 3 and 5) and isolated tissues in early postnatal development. RT‐qPCR revealed acute neuroinflammation in the DRGs, with expression levels of four inflammatory mediators elevated at P7, two at P13, and none at P21 in LPS‐treated rats. In contrast, the SC showed no change in inflammatory mediators at P7, elevation of two at P13 and four at P21 in LPS‐treated rats. These differences were greater in female SCs, indicating sex‐specific modulation even at these early stages of postnatal development. The SCs of P21 LPS‐treated rats also showed sex‐specific modulation of astrocytes (GFAP), with females showing an increase and males a decrease in GFAP. Together, these data indicate that during early postnatal development DRG neurons are more susceptible to acute inflammation whereas inflammation is delayed in the SC, with sex‐specific modulation occurring only in the SC.

## 1 INTRODUCTION

Early life insults can drive the expression of chronic disorders that persist well into adulthood. Clinical studies show premature infants exposed to painful stimuli in the NICU have increased responses to heat in early adolescence (Hermann et al., 2006). Similarly, preclinical work on early‐life pain in rodent models shows rat pups exposed to repeated paw pricks as neonates exhibit exaggerated responses to mechanical stimuli (Anand et al., 1999), and neonatal injection of the inflammatory agent carrageenan enhances pain responses in adult females (Laprairie & Murphy, 2007). Such long‐term changes can also be induced by early life inflammation. For example, exposure to lipopolysaccharide (LPS) in the first two postnatal weeks results in enhanced sensitivity to painful stimuli in adulthood (Boisse et al., 2005). Our previous work has validated these alterations in responses to noxious stimuli, with neonatal rats exposed to LPS exhibiting increased pain behaviors in response to formalin injection in adulthood (Zouikr et al., 2015). Most importantly, alterations in formalin‐induced pain behaviors emerge 1 week after neonatal LPS exposure, with postnatal day (P) 13 rats displaying increased licking following formalin injection (Zouikr et al., 2014). This finding was also observed in prenatally stressed rodents, with P7‐8 rats displaying enhanced behavioral responses to formalin injection compared to their non‐stressed counterparts (Butkevich et al., 2007). Together, these findings suggest early life inflammation can influence pain perception throughout life.

Although these changes in pain behaviors following neonatal inflammation are well documented, the underlying mechanisms remain elusive. The pain pathway begins in the periphery with the activation of nociceptors located on the peripheral axons of sensory neurons in the dorsal root ganglia (DRG). These signals subsequently enter the spinal cord dorsal horn where second‐order neurons pass signals along the pain neuroaxis to trigger pain perception and actions (pain behaviors) which reduce pain. Much of the information on neuroimmune responses in the DRG and spinal cord following noxious insults has been obtained in adult models (see (Kavelaars & Heijnen, 2021) for review). Few studies have examined the consequences of early life inflammation on first and second‐order neurons in the early postnatal and preadolescent preclinical models (Hathway et al., 2009; Vega‐Avelaira et al., 2012), and even fewer have considered potential sex‐specific modulation at these ages. Our previous work showed preadolescent rats exposed to neonatal LPS demonstrated increased flinching following formalin injection at P22 (Zouikr et al., 2014) as well as enhanced neuronal signaling and altered ionic currents in their spinal cord networks (Tadros et al., 2018). Thus, our data examining the pain behaviors of neonatal and preadolescent rats exposed to neonatal LPS implicate the DRG and spinal cord in altering pain behaviors during this early developmental period.

Given the complexity of neuroimmune interactions, modulation of key elements of this bi‐directional communication during critical periods of development is likely to underlie the long‐lasting alterations in pain behaviors following neonatal inflammation. Inflammatory mediators upregulated during early life inflammation are known to have a direct impact on spinal cord networks, with IL6 reducing inhibitory signaling, BDNF enhancing excitatory signaling (Lu et al., 2007), and IL1β influencing both inhibitory and excitatory transmission (Gustafson‐Vickers et al., 2008). Overall, this would result in an imbalance in synaptic connections, causing a net increase in signaling along the pain pathway. However, these discoveries were made in adults, and because there are notable differences in both the nervous and immune systems during these critical periods of development (Zengeler & Lukens, 2021), knowledge gained in adults may not apply to neonates. Accordingly, we have examined the profile of key inflammatory markers in both the DRG and spinal cord during the pre‐adolescent period following early life exposure to LPS.

## 2 MATERIALS AND METHODS

### 2.1 Animals

Experimental rats were derived by breeding naïve male and female Wistar rats (aged 10–12 weeks) acquired from the University of Newcastle's Animal House. Rat pups were obtained by mating one male with 4 females, and were sacrificed at either P7 (females *n* = 15; males *n* = 14), P13 (females *n* = 16; males *n* = 15), or P21 (females *n* = 16; males *n* = 14). These ages align with key changes in behavioral responses to inflammatory pain demonstrated previously by our group (Zouikr et al., 2013). Experiments were undertaken in accordance with federal and state legislation and as approved by the University of Newcastle Animal Care and Ethics Committee. All rats were maintained in a temperature (21°C ± 1) and humidity‐controlled environment on a 12 h light/dark cycle with food (Rat and Mouse Cube Chow, #SF00‐100, Speciality Feeds, Western Australia) and water available ad libitum for the duration of the study.

### 2.2 Intraperitoneal injection of lipopolysaccharide

On the day of birth (P1) all pups within a given litter were randomly allocated to receive either lipopolysaccharide (LPS) or saline (SAL) injections. Pups were briefly removed from dams on P3 and given an i.p. injection of either 0.05 mg/kg of LPS (pyrogen‐free saline and *Salmonella enterica*, serotype Enteritidis; Sigma Aldrich, #L2654) or SAL, and immediately returned to the dams. This process ensured pups were not isolated for more than 2 min, and was repeated on P5 and the pups were then left undisturbed with their dams until experimental timepoints of P7, 13, or 21 when they were sacrificed via guillotine for subsequent tissue collection and study. For each timepoint and tissue type (DRG or lumbar spinal cord) the experimental groups were as follows: female SAL, female LPS, male SAL, male LPS.

### 2.3 RNA extraction and RT‐qPCR

To quantify inflammatory markers in first and second order components of the pain pathway, thoraco‐lumbar dorsal root ganglia (T10 to L3; DRG) and the lumbar spinal cord (T12–L5) were removed following euthanasia. Both were immediately snap‐frozen to −80°C. To extract RNA, separate homogenates from a single representative DRG per animal and the lumbar region of the spinal cord were obtained. A sample of the resulting homogenate (10–20 mg) was used to assess total cellular RNA using the RNeasy® Mini Kit (Qiagen #74106, Hilden, Germany), according to the manufacturer's instructions. RNA concentration and quality were measured with a NanoDrop 1000 Spectrophotometer (ThermoFisher Scientific, USA).

Any contaminating genomic DNA within the RNA samples was digested using DNase I (Invitrogen #EN0523, Scoresby, Australia). Reverse transcription was then performed using Superscript III (Invitrogen #18080044, Scoresby, Australia), according to the manufacturer's instructions. Briefly, 30–200 ng of total RNA, 1 μL of oligo(dT)_18_ (Meridian Bioscience #BIO‐38029, Ohio, USA), 1 μL of random hexar (Meridian Bioscience #BIO‐38028, Ohio, USA), 1 μL of 10 μM dNTP (Meridian Bioscience #BIO‐65028, Ohio, USA), and molecular grade water to 13 μL, were mixed and heated for 5 min at 65°C in a Thermal Cycler (Eppendorf, Germany). After this step 4 μL of 5× first‐strand buffer, 1 μL of 0.1 M DTT (Meridian Bioscience #18080044, Ohio, USA), 1 μL RNaseOUT (40 U/μL) (Meridian Bioscience #BIO‐38028, Ohio, USA) and 1 μL SuperScript III RT (200 U/μL) were added and the mixture incubated for 60 min at 50°C, then 70°C for 15 min. Reverse transcription without Superscript III was also undertaken to determine the level of genomic DNA contamination.

All qPCR primers (Table 1) were designed with Ensembl using standard primer design criteria. The primer pairs were then screened through NCBI primer BLAST to ensure primer specificity. A total volume of 12.5 μL was used for the reaction containing: 6.25 μL 2× SensiFAST SYBR (Meridian Bioscience #BIO‐94020, Ohio, USA), 10 μM each of forward and reverse primers (Integrated DNA Technologies), 1–2.5 ng cDNA and molecular grade water to 12.5 μL. After an initial 10 min enzyme activation step at 95°C, 40 cycles at 95°C for 30 s (step 1) followed by 30 s at 60°C (step 2) were completed. Melt curves were generated to confirm the presence of a single PCR product. Primers were deemed specific if a single amplified product was detected by melt curve analysis. Reactions were performed on a 7500 Real Time PCR System (Applied Biosystems, USA) and analyzed using the Applied Biosystems 7500 Software (V2.3; Applied Biosystems, USA). For each primer the samples were run in triplicate, including a negative water control on each plate. Delta Ct (ΔCt, threshold cycle) was determined for each gene relative to the housekeeping genes β‐Actin and 18S. The ΔΔCt method (Livak & Schmittgen, 2001) was employed for comparisons between groups.

**TABLE 1:** Primer sequences.

Gene	Forward primer (5′–>3′)	Reverse primer (5′–>3′)
*Β‐Actin*	GCCCTAGACTTCGAGCAAGA	CAGGATTCCATACCCAGGAA
*18s*	CGCGGTTCTATTTTGTTGGT	CTTTCGCTCTGGTTCGTCTT
*IL‐6*	CCCAACTTCCAATGCTCTCCT	GGATGGTCTTGGTCCTTAGCC
*IL‐1β*	GCTACCTATGTCTTGCCCGT	AAGGTGCTTGGGTCCTCATC
*IL‐10*	TAAAAGCAAGGCAGTGGAGC	GTCACGTAGGCTTCTATGCAG
*BDNF*	ATTAGCGAGTGGGTCACAGC	TGGCCTTTTGATACCGGGAC
*IFN*	TGGAGGAACTGGCAAAAGGAC	TGTTGTTGCTGATGGCCTGG
*TNF⍺*	CGTCAGCCGATTTGCCATTT	CTCCAAAGTAGACCTGCCCG

### 2.4 Immunofluorescent labelling of microglia and astrocytes

P21 lumbosacral spinal cords (*n* = 4 per group) were dissected from euthanized rats and immersion‐fixed for 24 h in 4% paraformaldehyde (PFA) in phosphate buffer (PBS), then washed in PBS (3 changes). The cords were then washed in 80% ethanol (EtOH), dimethyl sulfoxide (DMSO) and 100% EtOH and embedded in 1450 MW polyethylene glycol (PEG). Transverse sections (20 μm thick) were cut on a rotary microtome and collected in PBS. Five sections from the lumbar enlargement (L2–L4) were collected and blocked in 10% normal donkey serum (NDS, Jackson ImmunoResearch). For labeling with antibodies against ionized calcium binding adaptor molecule 1 (Iba1; for microglia) and glial fibrillary acidic protein (GFAP; for astrocytes), sections were incubated overnight at room temperature in a solution of PBS with 0.1% Triton, 10% NDS, Iba1 (rabbit; 1:250; Wako #019‐19741), and GFAP (chicken; 1:1000; Merck Millipore #AB5541). After the primary incubation step, sections were rinsed in PBS and incubated for 2 h at room temperature with secondary antibodies 594‐donkey‐anti‐rabbit (1:50; Abcam #AB150076) and 488‐donkey‐anti‐chicken (1:50; Jackson ImmunoResearch #JI703545155) and NeuroTrace Blue (1:50, ThermoFisher Scientific #N21476). Sections were mounted on slides in buffered glycerol and cover slipped. Images were acquired at 10× magnification using an Olympus BX50 microscope equipped with a mercury burner and an Olympus DP72 camera. The superficial dorsal horn (SDH) was identified by comparing our Neurotrace Blue labeling with a spinal cord atlas (Watson et al., 2009) to generate a template for cropping which would be consistently applied to all images. Mean fluorescent intensity (MFI) was calculated independently for Iba1 and GFAP using ImageJ (National Institute of Health, USA). The selected image was converted into a 16‐bit image, duplicated and made binary. The binary image was redirected to the 16‐bit image and the particles were analyzed to reveal the MFI. This process was undertaken on all five images from each spinal cord, and an average was calculated for each rat. Images of Iba1‐positive cells were processed offline using a custom MATLAB script to analyze microglia number and morphology (Abdolhoseini et al., 2016, 2019; Kluge et al., 2017).

### 2.5 Data analysis and statistics

**FIGURE 1:**
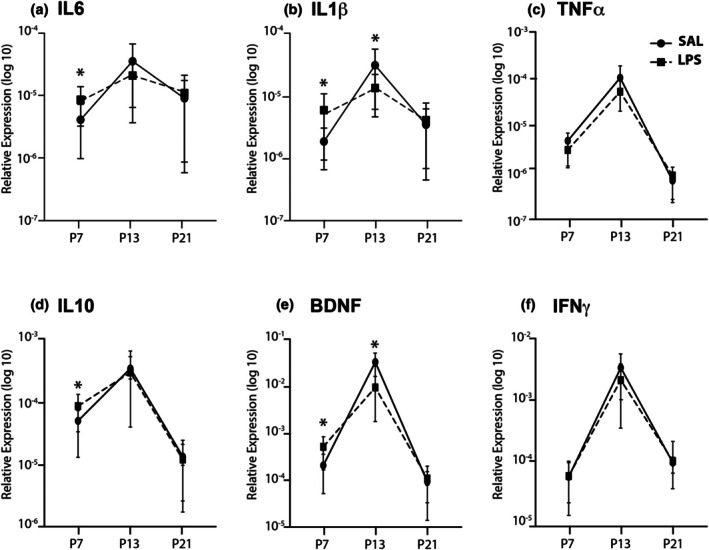
Developmental expression of key inflammatory mediators in the DRG following exposure to LPS. Line graphs of transformed data (mean ± SD) demonstrating age‐related changes in DRG of saline‐exposed rats (solid lines) compared to LPS‐exposed rats (dashed lines). At P7, LPS exposure resulted in an increase in IL6 (a), IL1β (b), IL10 (d), and BDNF (e), with no change in the relative expression of TNFα (e) or IFNγ (f). At P13, only IL1β (b) and BDNF (e) were decreased by LPS exposure, and no inflammatory mediators differed at P21. Developmentally, all mediators showed a peak at P13 in saline‐exposed animals, but this peak was not present for IL6 (a) or IL1β (b). *p < 0.05 for SAL (*n* = 14) versus LPS (*n* = 16).

**FIGURE 2:**
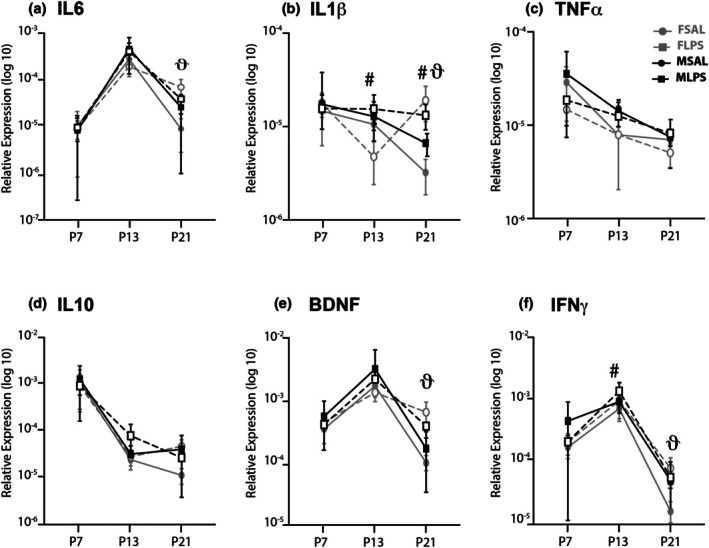
Developmental expression of key inflammatory mediators in the spinal cord following exposure to LPS. Line plots of transformed data (mean ± SD) demonstrating age‐related changes the spinal cord of saline exposed rats (solid lines) compared to LPS‐exposed rats (dashed lines) with males in black, and females in gray. There were no differences in any groups at P7, although increases in IL1β (b) and IFNγ (f) were observed in LPS exposed males at P13. At P21, neonatal LPS exposure resulted in a significant increase in IL1β (b) gene expression in both sexes. Females showed significant increases in gene expression of IL6 (a), BDNF (e) and IFNγ (f), with no changes in gene expression for males. # = *p* < 0.05 for male saline (*n* = 6) versus male LPS (*n* = 8), and ϑ = *p* < 0.05 for female saline (*n* = 8) versus female LPS (*n* = 7).

Data were analyzed via multivariate analysis of variance (ANOVA) using SPSS software (V25; IBM SPSS Statistics, New York, USA). All data in this study met the assumptions of normality and equality of variance. Outlying data points were rejected if they were more than 2 standard deviations from the mean. Post‐hoc comparisons were used to determine interaction effects and the Bonferroni correction was used for post‐hoc comparisons. All statistical analyses were conducted on untransformed data. To accommodate for large differences in relative expression across ages, the line graphs in Figures 1 and 2 show data that has undergone a log10 transformation in order to allow visual comparisons across age groups. Statistical significance was set at *p* ≤ 0.05.

## 3 RESULTS

### 3.1 Dorsal root ganglia

Neonatal exposure to LPS altered expression levels of key inflammatory mediators in DRGs (Figure 1). These changes were observed at individual time points as well as across the first 3 weeks of postnatal development. As no sex‐specific changes were observed in DRG samples, data for males and females were pooled.

At P7, expression levels for four inflammatory mediators were increased in the DRGs of LPS‐treated rats (Figure 1). Specifically, a significant effect of treatment was found for IL6 (*F*_(1,28)_ = 6.664, *p* = 0.016; Figure 1a), IL1β (*F*_(1,28)_ = 7.690, *p* = 0.01; Figure 1b); IL10 (*F*_(1,28)_ = 4.4152, *p* = 0.045; Figure 1d) and BDNF (*F*_(1,28)_ = 10.047, *p* = 0.004; Figure 1e) with all four markers shown to be elevated in rats exposed to LPS. At P13, only two genes were affected by LPS treatment, and in contrast to P7, their expression levels were lower than their SAL counterparts: IL1β (*F*_(1,27)_ = 6.892, *p* = 0.015; Figure 1b), and BDNF (*F*_(1,27)_ = 4.980, *p* = 0.035; Figure 1e). However, neonatal LPS exposure did not affect the expression of any markers in DRGs at P21. The expression levels of TNFα and IFNγ in the DRG were not affected by neonatal exposure to LPS at any of the ages examined. Together, these findings suggest neonatal exposure to LPS induces rapid changes and elevated expression of four key inflammatory genes in the DRG. However, these changes in gene expression do not persist past P21.

To assess the impact of LPS on the developmental trajectory of these key inflammatory mediators, we next compared the expression levels for each mediator over the three ages used in this study. In control rats there was a clear peak in gene expression in the DRG at P13 for all mediators: IL1β (*F*_(1,84)_ = 27.826, *p* < 0.001); IL6 (*F*_(1,84)_ = 7.710, *p* = 0.001); IL10 (*F*_(1,84)_ = 23.986, *p* < 0.001); TNFα (*F*_(1,84)_ = 28.906, *p* < 0.001); IFNγ (*F*_(1,84)_ = 50.323, *p* < 0.001) and BDNF (*F*_(1,84)_ = 36.017, *p* < 0.001). However, LPS exposure interrupted the normal developmental expression of IL6, IL1β, IL10, and BDNF (Figure 1). Specifically, the peak we observed in IL1β expression in control rats at P13 was not observed in LPS‐treated rats, although IL1β expression in both SAL and LPS rats decreased between P13 and P21 (age × treatment interaction: *F*_(1,84)_ = 8.542, *p* < 0.001; Post hoc for saline (*F*_(2,78)_ = 30.928, *p* < 0.001); Post hoc for LPS (*F*_(2,78)_ = 3.359, *p* = 0.04)). This altered developmental pattern suggests that LPS exposure induces a premature peak in IL1β expression. In addition, while saline‐treated rats displayed an increase in IL6 gene expression between P7 and P13, and a subsequent decrease between P13 and P21 (age × treatment interaction: *F*_(1,84)_ = 3.226, *p* = 0.045); Post hoc for saline (*F*_(2,78)_ = 10.063, *p* < 0.001), there was no significant effect of LPS treatment on age for IL6. This suggests neonatal LPS exposure flattens the developmental peak of IL6 expression by maintaining elevated levels throughout the first and second postnatal weeks. BDNF expression was elevated at both P7 and P13 following LPS exposure, and the increase in IL10 expression driven by LPS did not persist past P7. Overall, this suggests early life LPS exposure has the capacity to interrupt the normal developmental trajectory of these inflammatory mediators in the DRG during the early postnatal weeks.

### 3.2 Spinal cord

Neonatal exposure to LPS resulted in elevated expression of genes for several inflammatory markers in the spinal cord. Like the DRG, these changes occurred at specific time points during the first 3 weeks of postnatal development. However, in contrast to the DRG, sex‐specific changes in gene expression were observed. Accordingly, gene expression data are presented separately for male and female rats (Figure 2).

There was no effect of LPS on gene expression for any marker at P7 in both sexes (Figure 2). However, by P13, expression levels of two inflammatory mediators were impacted by neonatal LPS, and this occurred in a sex‐specific manner. Specifically, IL10 (*F*_(1,26)_ = 4.7586, *p* = 0.0391; Figure 2d) increased in LPS treated males compared to their male and female counterparts. IFNγ expression levels also increased, with greater responses in males relative to females (*F*_(1,26)_ = 6.854, *p* = 0.015; Figure 2f). At P21, neonatal LPS exposure induced greater and more complex changes in the gene expression levels of four key inflammatory mediators. LPS treated rats exhibited sexually dimorphic increases in the expression of IL6 (*F*_(1,27)_ = 20.808, *p* < 0.001; Figure 2a), IL1β (*F*_(1,27)_ = 33.692, *p* < 0.001; Figure 2b), BDNF (*F*_(1,27)_ = 12.141, *p* = 0.002; Figure 2e) and IFNγ (*F*_(1,27)_ = 8.819, *p* = 0.006; Figure 2f). These increases were greater in females, with increased expression of IL6, IFNγ and BDNF that were not observed in males (IL6‐treatment × sex interaction: *F*_(1,27)_ = 7.935, *p* = 0.009; Post hoc for females: *F*_(1,25)_ = 27.622, *p* ≤ 0.001; IFNγ‐treatment × sex interaction: *F*_(1,27)_ = 5.9181, *p* = 0.022; Post hoc for females: *F*_(1,25)_ = 15.635, *p* = 0.001; BDNF‐treatment × sex interaction: *F*_(1,27)_ = 12.141, *p* = 0.002; Post hoc for females: *F*_(1,25)_ = 19.136, *p* ≤ 0.001). Together, these data suggest LPS exposure does not immediately affect inflammatory mediator gene expression in either sex as it does in DRG. Rather, female rats exposed to LPS exhibit a delayed increase in key inflammatory markers 2 weeks after the initial inflammatory insult.

To further explore the impact of LPS exposure on the developmental trajectory of these key inflammatory mediators, we next compared the expression levels for each mediator across the three ages. In contrast to the DRG, only three of the six mediators demonstrated a clear peak in gene expression at P13 in control rats (IL6 (*F*_(2,84)_ = 53.648, *p* ≤ 0.001); IFNγ (*F*_(2,84)_ = 83.229, *p* < 0.001); and BDNF (*F*_(2,84)_ = 25.913, *p* < 0.001)). Expression of the remaining mediators, IL10, TNFα, and IL1β decreased from P7 to P21 without a discernible peak in the second postnatal week (IL10 (*F*_(2,84)_ = 57.406, *p* < 0.001); TNFα (*F*_(2,84)_ = 8.508, *p* < 0.001); IL1β (*F*_(2,84)_ = 3.056, *p* = 0.05)). The expression of IL1β and IFNγ was both developmentally modulated by neonatal LPS exposure, with levels of both mediators remaining high at P21 despite decreasing compared to P13 in saline‐treated rats (IL1β: age × treatment interaction: *F*_(2,84)_ = 4.474, *p* = 0.014; Post hoc for LPS: *F*_(2,78)_ = 3.684, *p* = 0.03; Post hoc for saline: *F*_(2,84)_ = 4.028, *p* = 0.022; IFNγ: age × treatment interaction: *F*_(2,84)_ = 4.555, *p* = 0.013; Post hoc for LPS: *F*_(2,78)_ = 65.606, *p* ≤ 0.001; Post hoc for saline: *F*_(2,78)_ = 24.806, *p* < 0.001). Together, this suggests LPS disrupts the normal developmental trajectory of key inflammatory mediators in the spinal cord, by modulating the balance of these key mediators across the first three postnatal weeks.

### 3.3 Spinal microglia and astrocytes

**FIGURE 3:**
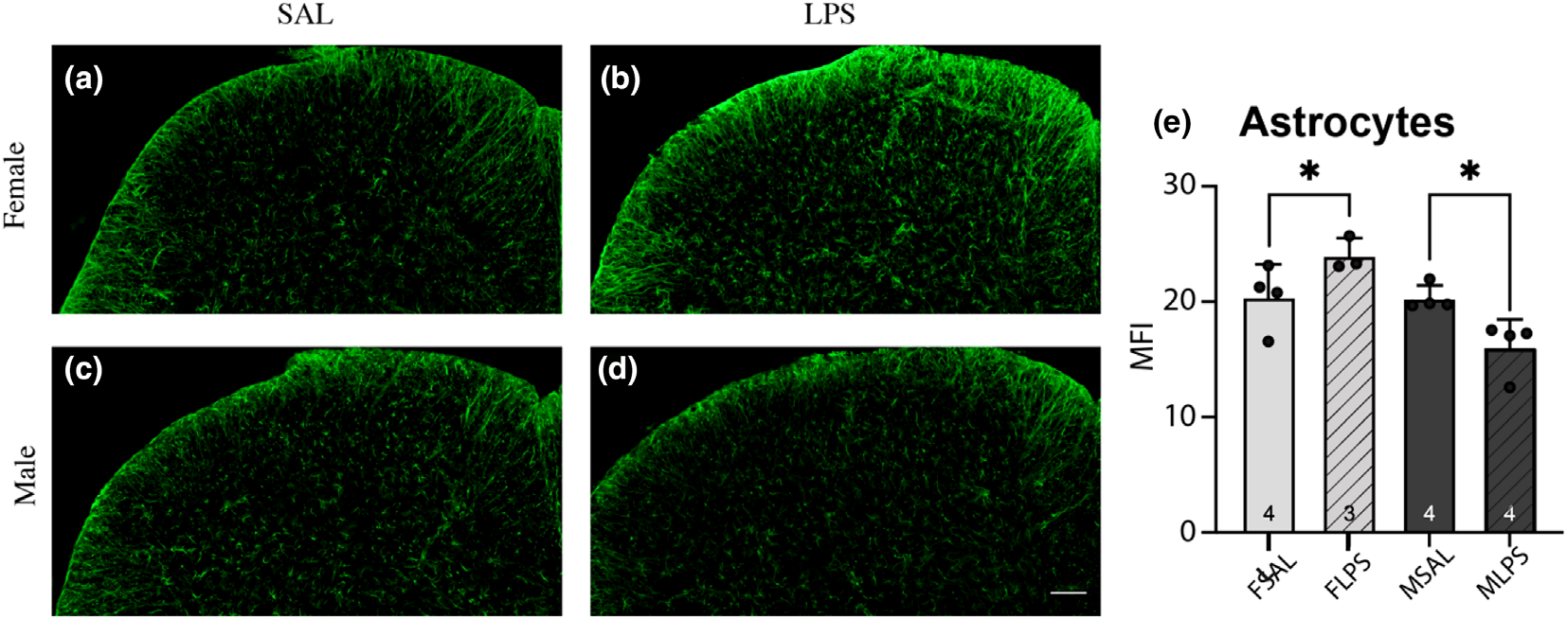
GFAP immunofluorescence in the superficial dorsal horn of the spinal cord at P21. Females exposed to neonatal LPS (b) showed significantly higher GFAP MFI (e) compared to saline‐injected controls (a), while males exposed to neonatal LPS (d) showed a significantly lower MFI (e) compared to their saline counterparts. Data are displayed as mean ± SD, with the total number of samples shown utilized to generate the mean in each bar * = *p* < 0.05.

**FIGURE 4:**
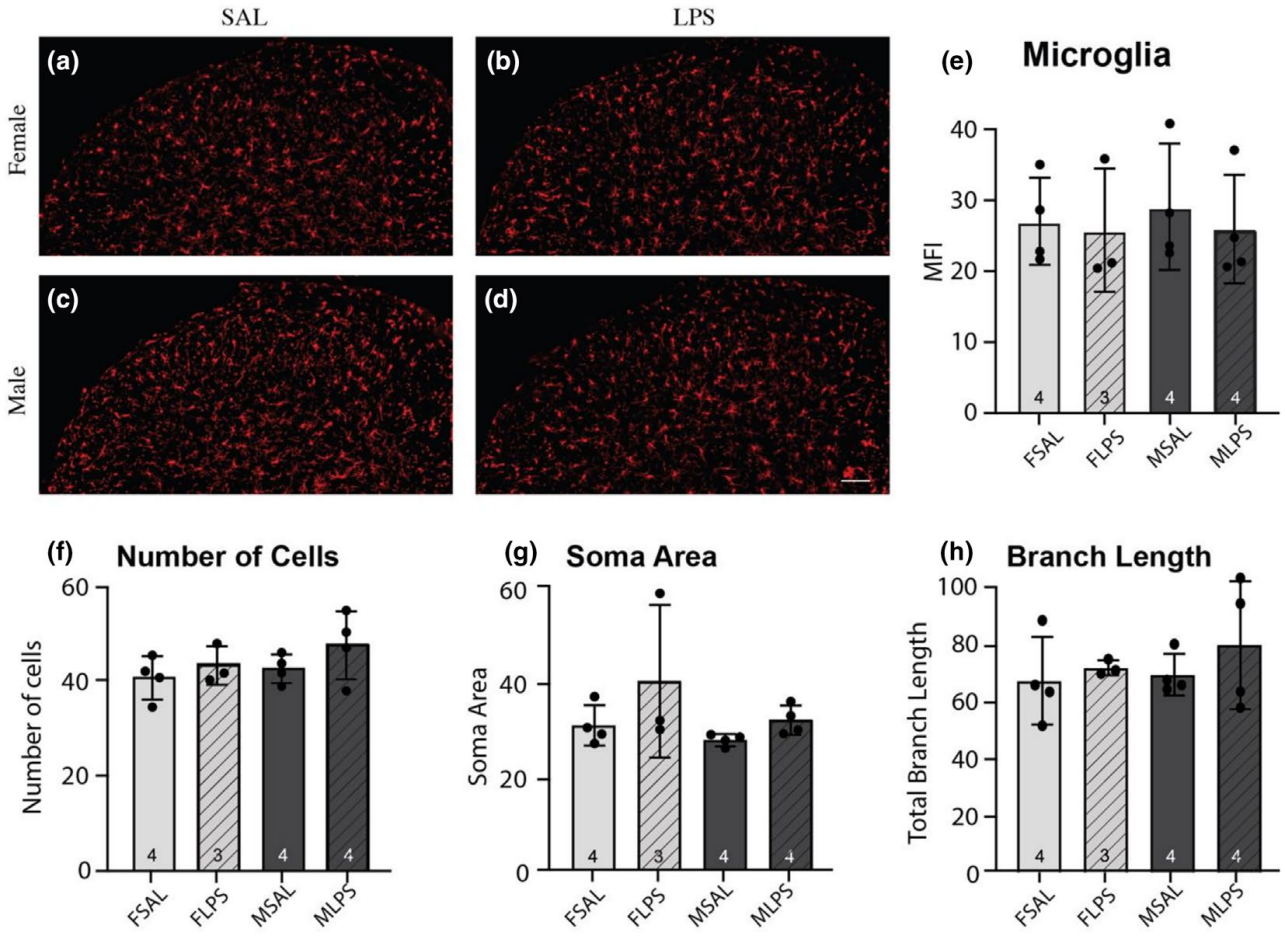
Iba1 immunofluorescence in the superficial dorsal horn of the spinal cord at P21. Mean fluorescent intensity (MFI) of Iba1 immunofluorescence (a–d) did not differ between the groups examined (e). Further, the number of Iba1‐positive cells did not differ between groups (f), nor did morphological analysis of soma area (g) or branch length (h). Data are displayed as mean ± SD, with the total number of samples shown utilized to generate the mean in each bar. * = *p* < 0.05.

Because the increase in inflammatory mediators in the spinal cord was greatest at P21 (Figure 2), we next examined astrocyte and microglia modulation after LPS exposure in the pain processing region of the spinal cord (i.e., the superficial dorsal horn, SDH; Figures 3 and 4). We observed modulation of GFAP fluorescent intensity and the nature of the change differed between sexes (sex × treatment interaction *F*_(1,14)_ = 12.892, *p* = 0.04; Figure 3e). GFAP mean fluorescent intensity increased in females after neonatal LPS exposure (Post hoc for females *F*_(1,11)_ = 5.071, *p* = 0.046), whereas it decreased in males (Post hoc for males *F*_(1,11)_ = 8.138, *p* = 0.016).

Surprisingly, there was no difference in the mean fluorescence intensity for Iba1 in any group (Figure 4). Given the importance of microglial morphology on activation status (Schwarz & Bilbo, 2012), we utilized a custom MATLAB script (Abdolhoseini et al., 2016; Kluge et al., 2017; Abdolhoseini et al., 2019) to undertake quantitative measures of cell number (Figure 4f); soma area (Figure 4g) and the branch length (Figure 4h) of Iba1‐positive cells. Although no values were significantly different between groups, we did note greater variability in the soma area of females exposed to LPS, and the branch length of males exposed to LPS, suggesting a more heterogeneous population of microglia is present in the SDH of rats exposed to neonatal LPS.

## 4 DISCUSSION

Our study demonstrates an acute upregulation of key inflammatory mediators in DRGs immediately following LPS exposure, which resolves within 2 weeks of the neonatal insult. This is followed by an increase in a similar suite of inflammatory mediators in the spinal cord in pre‐adolescent rats. Interestingly, these differences in the spinal cord are sex‐specific, with females showing a greater increase in gene expression of inflammatory mediators than males, even though these rats are pre‐adolescent. Furthermore, these sex‐specific differences are matched by sex‐specific modulation of the immunoreactivity of astrocytes in the superficial dorsal horn, with females showing increases while males exhibit decreased GFAP levels. These data emphasize the need for sex‐specific studies of neuroimmune interactions throughout the developmental period, at all nodes of the pain pathway in order to elucidate the mechanisms underlying the long‐term impacts of neonatal immune activation.

### 4.1 Evidence for an indirect impact on spinal cord networks

We observed significant interactions between age and treatment in both the DRG and spinal cord, suggesting neonatal LPS exposure interrupts the normal developmental trajectory of these two critical regions in the pain neuroaxis. For the DRG, the inflammation observed at P7 appeared to resolve within 2 weeks of LPS exposure, with no differences in the inflammatory profile at P21. In contrast, we did not observe changes in the P7 spinal cord, but found significant evidence of increased inflammation at P21, that is, 2 weeks after the initial LPS insult. This suggests a direct impact of LPS on peripheral DRG, but an indirect mechanism for alterations in spinal networks. Moreover, these interactions were also observed in a sex‐specific manner in the spinal cord, with females displaying greater deviation from the saline controls than males.

Intriguingly, our findings differ from prior studies using neonatal LPS insults, with prior studies reporting increases in the expression of key inflammatory mediators in the spinal cord within 24 h of an injection of LPS at similar developmental timepoints (Hunter et al., 2014; Hsieh et al., 2018). However, both prior studies utilized only a single injection of LPS at much higher concentrations and examined cytokine levels within 24 h of exposure, whereas we utilized two injections at P3 and P5 and documented modulation of cytokine levels 2 weeks after the initial insult. It is well known that the exact doses, timing and ages of LPS exposure influence the final outcome (Bao et al., 2022), subsequently it is hard to make a direct comparison between different applications of LPS during this critical window of development. Moreover, while Hsieh et al. did compare the sexes and found no difference between males and females in their study, Hunter et al. do not report any sex‐specific considerations at all. Therefore, the sex‐specific modulation we observed may have been obscured by the combination of both sexes in the prior studies.

Cytokines are known to be essential in neurodevelopment (see (Zengeler & Lukens, 2021) for review), with many interleukins and growth factors implicated in neurogenesis, differentiation and even the formation and maintenance of synapses. Moreover “critical periods” during development exist for the above processes. Thus, the role these cytokines play in neurodevelopment is complex, and highly dependent upon the developmental stage, microenvironment and activation of signaling pathways. The modulation of key inflammatory mediators observed in this study occurred over known critical periods in spinal cord development (Walsh et al., 2009) during which expression of certain types of potassium channels that contribute to neuronal excitability changes markedly. These potassium channels are known to be modulated by elevated cytokines arising from maternal immune activation in CA1 hippocampal neurons (Griego et al., 2022), suggesting a complex relationship exists between the altered cytokine levels and ongoing neurodevelopment following neonatal inflammation.

An overriding contributor to the impact of a peripheral inflammatory agent, like LPS, is the blood‐brain barrier (BBB). The BBB protects the spinal cord, but not the DRG, which is protected by a different structure known as the blood‐DRG barrier, known to have less tight junctions and more capillaries than the BBB (BDB; Reinhold & Rittner, 2020). Interestingly, the BBB has been shown to be *more effective* during early development (Saunders et al., 2012), suggesting that the LPS exposure in our model would be unlikely to cross the BBB and therefore, the inflammatory profile we observed would be due to some other mechanism. Although only examined in adult preclinical models, the BDB shows downregulation of tight junction proteins following chronic constriction injury which is hypothesized to facilitate invasion by immune cells, including macrophages known to secrete cytokines (Lux et al. 2020). The development of the BDB remains understudied; however, its known increased permeability could be responsible for allowing LPS to have a more direct impact on inflammatory mediators within the DRG.

### 4.2 The role of glia in the long‐term impact of early life inflammation

Interestingly, we observed no change in Iba1 immunofluorescence in either sex at P21, suggesting microglia are not heavily involved in the altered levels of inflammatory mediators we observed in the female spinal cord. This is surprising, given microglia in the brain have been shown to react strongly to early life stress (for review see; Schwarz & Bilbo, 2012), and it could be due to our small sample size. We did note an increase in variability in microglia morphological properties of rats exposed to LPS, which could account for some of the neuroimmune modulation observed at P21. However, astrocytes can also be influenced by peripheral inflammation to produce a suite of inflammatory mediators, which can be either pro‐inflammatory and neurodestructive, or anti‐inflammatory and neuroprotective (see (Colombo & Farina, 2016) for review). In our sample, we observed a concurrent increase in both pro‐inflammatory cytokines IL6 and IL1β and anti‐inflammatory mediators IFNγ and BDNF in the spinal cord of P21 females exposed to neonatal LPS, as well as an increase in GFAP immunofluorescence. In contrast, there were no differences in these inflammatory mediators in male P21 spinal cords, and GFAP immunofluorescence decreased. Therefore, it is plausible that female spinal astrocytes are modulated by early life LPS exposure and are implicated in the altered levels of inflammatory mediators. More work is needed to determine whether this modulation is neuroprotective or destructive.

In naïve animals, male cortical astrocytes reach a “mature” phenotype at P7, while female astrocytes reach the same level of maturity later at P14 (Rurak et al., 2022). While sex‐specific alterations of glia within the brain are known to occur in a number of laboratory models of early life inflammation (for review see; Schwarz & Bilbo, 2012), few studies have examined the impact on glia within the spinal cord. Studies in models of CNS injury in adult animals have revealed diverse responses of microglia in the spinal cord compared to the brain (for summary see: Sheilds et al., 2020), which suggests developmental differences could also exist. Spared nerve injury on male rats during early postnatal development (P0–P21) impacts spinal cord astrocytes more heavily than microglia (Vega‐Avelaira et al., 2007). Moreover, this study also showed that upregulation of GFAP was delayed in younger animals—changes were not observed until 5 days after an injury inflicted during the first postnatal week. Similarly, the impact of neonatal anoxia on sensorimotor processing demonstrated increased GFAP, as measured by western blot, in the spinal cord of adult female rats exposed to neonatal anoxia. Importantly, this aligned with a decrease in mechanical and thermal thresholds for paw withdrawal (Helou et al., 2021). Interestingly, although adult males exposed to neonatal anoxia also displayed decreased mechanical thresholds, there was no change in their spinal cord GFAP levels. Together with the findings of this study, these data suggest astrocytes are more likely to show long‐term modulation following neonatal inflammation, and this modulation occurs in a sex‐specific manner.

### 4.3 Implications for pain behaviors

Inflammatory mediators are known to have a direct impact on neuronal signaling, with many cytokines exerting an influence on specific elements of neuronal communication. Within spinal cord networks, IL6 reduces inhibitory signaling and BDNF enhances excitatory signaling (Lu et al., 2007). Furthermore, IL1β causes concurrent increases in excitatory and decreases in inhibitory signaling (Gustafson‐Vickers et al., 2008) and an imbalance and overall increase in the excitation of spinal cord networks. This is important because enhanced spinal cord excitation would increase traffic along the entire pain pathway, ultimately enhancing the signal reaching the brain and impacting pain perception and behaviors. At P21, we observed a significant increase in IL6, IL1β, and BDNF in the spinal cord of females exposed to neonatal LPS, suggesting enhanced signaling exists in the spinal neuronal network of these animals. Indeed, our previous work shows increased excitatory currents within spinal cord networks at P22 (Tadros et al., 2018), as well as heightened flinching in P22 rats following neonatal LPS (Zouikr et al., 2014). Together, our work on the spinal cord of preadolescent rats exposed to neonatal LPS suggests the neural processing within spinal cord networks plays a role in altering pain behaviors following early life inflammation.

Sex‐differences in pain conditions are widely described in the literature (see (Mogil, 2012) for review); however, the evidence for sex‐specific modulation of spinal cord neural processing is less clear. Research examining the impact of neonatal hind paw incision, a laboratory model that mimics neonatal surgery, demonstrated a reduction in inhibitory signaling within neural networks of adult spinal cords exposed to the neonatal incision. However, there were no differences between the modulation observed in male and female adults (Li & Baccei, 2019). Follow‐up work in adult female mice exposed to the neonatal hind paw incision also demonstrates an imbalance in excitatory/inhibitory connections within a subpopulation of spinal cord neurons (Brewer et al., 2020), however similar experiments were not carried out in males, so it is unclear whether this particular imbalance is sex‐specific. Clearly, spinal cord networks and their associated processing of nociceptive signals are susceptible to early life interventions, and the data in the present study highlight the need to examine changes at multiple stages of development, not just in adulthood.

Together, our findings provide evidence of an indirect impact of neonatal LPS on spinal cord networks, with sex‐specific modulation occurring prior to adolescence. We found evidence of altered inflammation in the spinal cord at P21, which was the latest age examined in this study. Additionally, these changes did not emerge until 2 weeks after the initial LPS insult, implicating an indirect mechanism in this modulation of inflammatory mediators. Alongside these altered inflammatory mediators, we also observed sex‐specific modulation of astrocytes at P21, which could contribute to the activation of both neurodestructive and neuroprotective pathways. This corroborates the concept of “priming,” whereby early life insults alone may not result in an overtly altered phenotype; however, it may increase the impact of a second insult later in life. This underlies the development of two‐hit laboratory models which demonstrate an exaggerated response in adults pre‐exposed to LPS during early development (e.g., Zhao et al., 2008; Tufvesson‐Alm et al., 2020). This highlights the importance of examining both sexes during the early postnatal developmental period to fully elucidate the role of early life events in shaping future pain behaviors.

## FUNDING INFORMATION

Prof. Deborah Hodgson reports that financial support was provided by the National Health and Medical Research Council.

## CONFLICT OF INTEREST STATEMENT

The authors declare they have no competing interests.

## Data Availability

The data that support the findings of this study are available from the corresponding author upon reasonable request.
